# Psychological and Physical Health of a Preterm Birth Cohort at Age 35 Years

**DOI:** 10.1001/jamanetworkopen.2025.22599

**Published:** 2025-07-22

**Authors:** Amy L. D’Agata, Charles Eaton, Tara Smith, Dorothy Vittner, Mary C. Sullivan, Douglas A. Granger, Bing Lu, Justin Parent

**Affiliations:** 1 College of Nursing, University of Rhode Island, Kingston; 2 Department of Family Medicine, Warren Alpert Medical School of Brown University, Providence, Rhode Island; 3 Department of Epidemiology, Brown University School of Public Health, Providence, Rhode Island; 4 Center for Primary Care and Prevention, Kent Hospital, Pawtucket, Rhode Island; 5 Egan School of Nursing & Health Studies, Fairfield University, Fairfield, Connecticut; 6 Department of Psychological Science, University of California at Irvine; 7 Department of Pediatrics, Johns Hopkins University School of Medicine, Baltimore, Maryland; 8 Department of Public Health Sciences, University of Connecticut Health Center, Farmington; 9 College of Health Sciences, University of Rhode Island, Kingston

## Abstract

**Importance:**

The US’s longest-running preterm birth cohort study is nearing its 40th anniversary. With rising survival rates for preterm birth, understanding adult health outcomes is essential; early life medical risk trajectories are hypothesized to lead to poorer outcomes by age 35 years.

**Objective:**

To examine how early life medical risk is associated with psychological and physiological health in adulthood, highlighting the supportive roles of social protection and childhood socioeconomic status (SES).

**Design, Setting, and Participants:**

This cohort study included data from a longitudinal follow-up of a cohort from a level III neonatal intensive care unit in New England between 1985 and 1989, along with a control group of healthy full-term infants. Data were collected from March 2020 to March 2024 for the tenth follow-up using a longitudinal cohort approach. Inclusion criteria targeted preterm infants weighing under 1850 g with various neonatal diagnoses. Critically ill infants with low survival or major congenital anomalies were excluded. Biospecimens, physiological measurements, and imaging data were collected at a single clinical facility, while self-report surveys were gathered at home.

**Exposure:**

Early life medical risk from preterm birth.

**Main Outcomes and Measures:**

Primary outcomes included psychological and physiological health. Adult self-report questionnaire, blood pressure, cholesterol levels, triglycerides, glucose, glycosylated hemoglobin, insulin resistance, and dual-energy x-ray absorptiometry scan were measured. Statistical analyses included latent growth curve and path models using the medical risk index, social protection index, and SES.

**Results:**

The study sample included 158 preterm and 55 full-term–born adults (mean [SD] age, 35.0 [1.3] years; 107 [50.2%] female). Higher medical risk severity was associated with increases in internalizing problems (β [SE], 0.85 [0.33]; *P* = .01), higher systolic blood pressure (β [SE], 7.15 [2.47]; *P* = .004), lower high-density lipoprotein cholesterol (β [SE], −13.07 [4.4]; *P* = .003), higher triglycerides (β [SE], 53.97 [24.6]; *P* = .03), higher android-to-gynoid fat ratio (β [SE], 0.22 [0.08]; *P* = .006), and lower bone density (β [SE], −1.14 [0.40]; *P* = .004).

**Conclusions and Relevance:**

In this cohort study, preterm individuals had higher early life medical risk and faced increased mental health disorders, cardiometabolic issues, and body composition differences compared with full-term peers at age 35 years. Despite strong evidence linking preterm birth to long-term health consequences, many primary care clinicians in the US remain unaware of these risks, often due to infrequent birth history inquiries in adult health care settings.

## Key Points

**Question:**

How do early life medical risk factors for individuals born preterm compare with those born full-term in projecting adult psychological and physiological trajectories?

**Findings:**

In this cohort study using a longitudinal follow-up of a US birth cohort at age 35 years, greater early life medical risk severity was significantly associated with higher internalizing mental health issues, blood pressure, triglycerides, and body fat distribution, along with lower high-density lipoprotein cholesterol and bone density.

**Meaning:**

These results suggest that as the preterm-born population ages, it is increasingly important to understand their specific health needs to optimize health outcomes and health care resources.

## Teaser

This cohort study including data from 35-year follow-up on a US birth cohort examines the association of preterm birth with psychological and physical health trajectories.

## Introduction

The longest continuously running US study of individuals born preterm is approaching its 40th anniversary since initial recruitment.^1^ The RHODE (Rhode Island Cohort of Adults Born Preterm) Study cohort, born in the 1980s, emerged when survival steadily improved for infants weighing less than 1.5 kg.^2,3^ Altered neurodevelopmental trajectories during childhood, heightened prevalence of psychological problems in adolescence, and increased risk of cardiovascular disease in young adulthood have been key findings among individuals born preterm in this cohort.^4,5,6^ As part of the tenth follow-up study, we comprehensively assessed health and function in the third decade of life (a historical overview of the RHODE Study is available in eTable 1 in Supplement 1).

When considering the global magnitude of preterm birth, since 1980, approximately 500 million babies around the world have been born preterm.^7,8,9^ Despite a portion likely not having survived their preterm birth, this figure exceeded the entire 2024 US population. Among adults navigating the US health care system, a crude estimate is that about 8 million of them were born preterm^8^; thus, it is imperative to understand the health status of this population to ensure quality of care.

A long-held misconception posits that prematurity is exclusively an issue of infancy, with its effects diminishing by early childhood. This belief has been dispelled by contemporary research, which demonstrates that preterm birth results in lifelong consequences, effectively categorizing preterm birth as a chronic condition.^10,11,12,13^ According to international studies, adults born preterm face an increased risk of various chronic conditions, including diabetes, heart failure, ischemic heart disease, kidney disease, and stroke.^14,15,16,17,18^

A paradigm shift in healthcare is imperative. The US preterm birth rate has remained at 10% to 12% for decades, yet misconceptions claim the effects of prematurity only matter in childhood. This misunderstanding perpetuates myths among clinicians, educators, families, and those affected. By viewing prematurity as a lifelong condition, health care systems can better allocate resources and develop targeted interventions that support individuals from infancy to adulthood.

Much of the existing research on individuals born preterm comes from international studies focusing on highly homogeneous populations and national health care systems. There is an urgent need to investigate health outcomes, specifically in a US-born cohort of adults who were born preterm. As health care costs soar and US preterm survival rates continue to rise, we must deepen our understanding of later life health outcomes to develop targeted interventions that improve health trajectories and guide changes in health policy and clinical practice.

This is the first US-based investigation to characterize psychological and physical health trajectories from birth to the third decade. We analyze how risk and protective factors may affect psychological and physiological maladjustment trajectories in adulthood. Our primary independent variables are a medical risk index derived from a multi-informant, multimethod assessment of early life risk factors from birth to age 12 years.^19^ We also explore the role of social protective factors in mitigating risk trajectories and the association of socioeconomic status (SES) with outcomes. We hypothesize that early life trajectories from preterm birth and higher medical risk will be associated with poorer outcomes at age 35 years.

## Methods

### Design and Setting

The RHODE study applied a longitudinal cohort design to collect prospective data from participants in their third decade of life. Researchers collected data at a clinical research facility and participants’ homes. The clinical research facility obtained comprehensive data, including biospecimens, physiological measurements, imaging studies, performance-based tasks, and survey responses. Participants completed self-report demographic surveys (eg, race) and questionnaires in the home setting. Some of the selected measures were repeated assessments, whereas others were standard evaluations of overall health. All study participants were requested to attend a single in-person clinic visit (additional details available in eMethods in Supplement 1). A cash incentive was given. The University of Rhode Island’s institutional review board approved the study, coordinated activities, and managed data under a SMART agreement with the clinical research facility and assuming responsibility for data management. Participants or their guardians provided written informed consent. This report complies with Strengthening the Reporting of Observational Studies in Epidemiology (STROBE) reporting guideline.

### Sample

The study cohort consisted of 215 infants recruited from a level III neonatal intensive care unit (NICU) in New England from 1985 to 1989, utilizing recruitment methods previously described in detail (eTable 1 in Supplement 1).^4,20^ Inclusion criteria focused on preterm infants with birth weights under 1850 g and various neonatal diagnoses, excluding those who were critically ill with low survival probabilities or significant congenital anomalies. Recruitment also included a control group of healthy, normal-weight, full-term infants. Eligibility for mothers required good health, a minimum age of 16 years, and proficiency in English; mothers with intellectual disabilities or mental illness were excluded. The sample had an equal sex distribution and balanced SES. Following the deaths of 2 participants before this study’s commencement, the maximum sample size was adjusted to 213 from the original 215.

### Measures

#### Psychological Health

The ASEBA (Achenbach System of Empirically Based Assessment) is the most widely used measure of internalizing and externalizing problems.^21^ Adult Self-Report (ASR) was assessed in both the 23-year and 35-year follow-up studies; Youth Self-Report (YSR) was used in the 17-year follow-up study.^22^ At each time point, broadband internalizing and externalizing scales were utilized. Internalizing scale uses subscales of anxious and/or depressed, withdrawn, and somatic complaints. Externalizing scale uses subscales of aggressive behavior, rule-breaking behavior, and intrusiveness. Reliability for internalizing at age 23 years was ω = 0.92 and at age 35 years was ω = 0.95; externalizing at 23 years was ω = 0.87 and at age 35 years ω = 0.90 (details on scoring in eMethods in Supplement 1).

#### Blood Pressure

After 5 minutes of seated rest using an appropriate cuff, a research nurse took blood pressure measurements 3 times using a calibrated manual aneroid sphygmomanometer in at least 5-minute intervals. The 3-reading average was used.

#### Blood Specimens

After at least 9 hours of fasting, blood was collected by a certified phlebotomist at the clinical site. Tests included total cholesterol, high-density lipoprotein (HDL), low-density lipoprotein (LDL), triglycerides, glycosylated hemoglobin, glucose, and insulin resistance. Blood specimens were processed and stored in a −80 °F freezer at the clinical site until batch shipment at the end of the study (for assay methods, see eMethods in Supplement 1).

#### Dual-Energy X-Ray Absorptiometry Scan

Body fat distribution and bone density imaging were obtained through dual x-ray absorptiometry (DEXA scan) (GE Healthcare). Ratio of android fat mass to gynoid fat mass (android fat / gynoid fat) was calculated. Bone density was calculated using T scores and *z* scores based on age for the bone mineral content of the arms, legs, trunk, android, and gynoid regions. Prior to DEXA scan tests, all female participants underwent urine testing for human chorionic gonadotropin to check for pregnancy. If confirmed, imaging was postponed until the participant was no longer pregnant.

#### Medical Risk Index

Cumulative medical risk was indexed across multiple follow-up assessments. Birth variables included the Hobel neonatal illness acuity score,^23^ length of hospital stay, birth weight, and gestational age. Neonatal medical variables included medical health status, necrotizing enterocolitis, bronchopulmonary dysplasia, and total hours of supplemental oxygen. Neonatal neurological variables included neurological health status, intraventricular hemorrhage, and shunted hydrocephalus. Medical risk at toddlerhood and childhood included medical and neurological health status variables at age 18 and 30 months and ages 4, 8, and 12 years (eMethods in Supplement 1).

#### Social Protection Index

Proximal social factors were indexed from multiple assessments in the home, maternal involvement, and maternal control. Cognitive stimulation and emotional support within the home environment were measured using direct observation and semi-structured maternal interviews with the Home Observation for Measurement of the Environment^24^ (HOME) instrument during home visits at birth and ages 4, 8, and 12 years (eMethods in Supplement 1).

#### Family SES

Socioeconomic risk was assessed at each time point using the Hollingshead Four-Factor Index.^25^ Social status was based on 4 domains: marital status, retired or employed status, educational attainment, and occupational prestige. There were no significant differences in SES over time; therefore, the present study used childhood SES.

### Statistical Analysis

Primary analyses were split into 2 methods based on the number of waves available. First, when 3 waves (ages 17, 23, and 35 years) of the same measure were collected, we used latent growth curve models in Mplus version 8.8 (Muthén and Muthén) to capture intraindividual change over time. Given that our primary interest was in the trajectory and levels at age 35 years, we used a reverse slope, allowing for associations with levels at age 35 years and the trajectory from age 17 to 35 years. When 3 waves were unavailable due to measures not being collected at the last 3 waves, we used a path analysis model with health outcomes at age 35 years. The same 3 independent variables were used for all models—the medical risk index, social protection index, and childhood SES, all assessed from birth to age 12 years. The medical risk index was the primary variable, with social protection and childhood SES serving as contextual covariates. Following an inspection of missing data patterns, full information maximum likelihood (FIML) was used to include all available data. Statistical significance was defined as *P* < .05 across all models.

## Results

### Enrollment

A total sample of 158 preterm and 55 full-term born adults (mean [SD] age, 35.0 [1.3] years; 107 female [50.2%]; 17 Black [8.0%], 9 Hispanic [4.2%], 186 White [87.3%]) were included in the study analysis (Table 1). We examined the differences between preterm and full-term groups on key demographic variables using independent sample *t* tests for continuous variables and χ^2^ tests for categorical variables. Sex distribution, age, and race were similar in the 2 groups. However, the 2 groups differed in childhood SES (*t*[122] = −2.16; *P* = .03; *d* = −0.50), such that the full-term group had lower childhood SES; thus, childhood SES was controlled for in all analyses. Preterm infants weighed between 640 and 1820 g and were born at gestational ages ranging from 24 to 36 weeks. Descriptive characteristics of the preterm and full-term born groups at age 35 years are presented in eTable 2 in Supplement 1; bivariate correlations are presented in eFigure in Supplement 1. We examined missing data patterns at the 35-year wave (143 participants retained at age 35 years) by exploring demographics or outcomes at prior waves as indicators of dropout. No significant associations with dropout emerged, suggesting data were missing at random and appropriate for FIML missing data methods for inclusion of the full sample in analyses.

**Table 1.** Cohort Demographics at Birth

Characteristic	Individuals, No. (%)	
	Preterm (n = 158)	Full-term (n = 55)
Maternal age, mean (SD), y	27.2 (5.7)	26.0 (4.8)
Paternal age, mean (SD), y	29.8 (6.5)	28.9 (6.1)
Sex		
Male	79 (50.0)	27 (49.1)
Female	79 (50.0)	28 (50.9)
Race and ethnicity		
Asian	1 (0.6)	0
Black	13 (8.2)	4 (7.3)
Hispanic or Latino	5 (3.2)	4 (7.3)
White	139 (88.0)	47 (85.5)
Family status		
Single	23 (14.8)	5 (9.1)
Cohabitating	11 (7.1)	8 (14.5)
Married	121 (78.1)	42 (76.4)
Maternal education		
Partial HS	25 (16.2)	6 (11.1)
HS or GED	41 (26.6)	20 (37.0)
Partial college	23 (14.9)	11 (20.4)
College	64 (41.6)	15 (27.8)
Graduate degree or higher	1 (0.6)	2 (3.7)
Paternal education		
Partial HS	18 (12.2)	7 (13.0)
HS or GED	46 (31.1)	20 (37.0)
Partial college	13 (8.8)	6 (11.1)
College	67 (45.3)	20 (37.0)
Graduate degree or higher	4 (2.7)	1 (1.9)
Family SES		
High	33 (20.9)	7 (12.7)
Moderate high	40 (25.3)	16 (29.1)
Average	36 (22.8)	17 (30.9)
Moderate low	26 (16.5)	10 (18.2)
Low	23 (14.6)	5 (9.1)
Medical risk		
Bronchopulmonary dysplasia	31 (19.6)	0
Necrotizing enterocolitis	17 (10.8)	0
Sepsis	18 (11.4)	0
Meningitis	4 (2.5)	0
IVH		
No IVH	122 (77.2)	55 (100)
Grade I	11 (7.0)	0
Grade II	9 (5.7)	0
Grade III	10 (6.3)	0
Grade IV	6 (3.8)	0
Duration of oxygen, mean (SD), h	402.9 (670.0)	0
Hobel neonatal score, mean (SD)	86.2 (31.9)	1.5 (3.5)
Hobel birth score, mean (SD)	140.8 (36.4)	13.5 (12.7)
Birth weight, mean (SD), g	1271.6 (324.9)	3419.5 (394.8)
Gestational age, mean (SD), wk	30.0 (2.7)	39.8 (0.9)
Length of stay, mean (SD), d	52.4 (26.8)	3.0 (0.9)

Abbreviations: GED, General Educational Development accreditation; HS, high school; IVH, intraventricular hemorrhage; SES, socioeconomic status.

### Psychological Health

Model fit ranged from acceptable to excellent across models (eTable 3 in Supplement 1). We examined longitudinal associations between medical risk, social protection, and childhood SES from birth to age 12 years with internalizing and externalizing trajectories across the 17-, 23-, and 35-year waves. Higher birth-childhood medical risk severity was associated with increases in adulthood internalizing problems (β [SE], 0.85 [0.33]; *P* = .01), but was not associated with externalizing trajectories (Table 2). Figure demonstrates the form of this association by plotting estimated slopes for average medical risk levels for children born full-term and preterm. Neither social protection nor childhood SES was associated with internalizing or externalizing problems.

**Table 2.** Primary Outcome Results

Factors	Medical risk index (0-12)		Social protection (0-12)		Family SES (0-12)	
	β (SE)	*P* value	β (SE)	*P* value	β (SE)	*P* value
**Model 1** ^a^						
Internalizing						
35-y Level (intercept)	6.31 (4.08)	.12	−0.22 (0.21)	.30	−0.08 (0.09)	.36
Adulthood trajectory (slope)	0.85 (0.33)	.01	−0.01 (0.02)	.63	−0.01 (0.01)	.49
Externalizing						
35-y Level (intercept)	−0.33 (3.74)	.93	−0.24 (0.19)	.20	−0.04 (3.74)	.93
Adulthood trajectory (slope)	0.25 (0.31)	.41	−0.01 (0.02)	.60	0.00 (0.01)	.95
Systolic blood pressure						
35-y Level (intercept)	7.15 (2.47)	.004	0.16 (0.15)	.26	−0.06 (0.07)	.36
Adulthood trajectory (slope)	−0.12 (0.20)	.56	0.01 (0.01)	.85	−0.01 (0.01)	.25
Diastolic blood pressure						
35-y Level (intercept)	3.91 (3.13)	.21	−0.16 (0.20)	.44	0.01 (0.07)	.95
Adulthood trajectory (slope)	0.20 (0.35)	.56	−0.02 (0.02)	.42	0.01 (0.01)	.89
**Model 2** ^a^						
Cardiovascular health						
HDL cholesterol	−13.07 (4.4)	.003	0.41 (0.23)	.08	0.10 (0.12)	.42
LDL cholesterol	9.47 (9.87)	.34	−0.23 (0.46)	.61	−0.13 (0.24)	.59
Metabolism and energy						
Triglycerides	53.97 (24.6)	.03	−0.39 (0.86)	.65	−0.20 (0.51)	.69
Hemoglobin A_1c_	0.10 (0.17)	.54	−0.01 (0.01)	.31	−0.01 (0.01)	.33
Inflammation and immune						
C-reactive protein	2.03 (2.23)	.36	−0.02 (0.12)	.84	−0.09 (0.05)	.09
Interleukin-6	0.70 (0.67)	.29	−0.02 (0.04)	.66	−0.04 (0.02)	.01
DEXA scan						
Android-gynoid fat ratio	0.22 (0.08)	.006	0.00 (0.01)	.99	−0.01 (0.02)	.82
Bone density T score	−1.14 (0.40)	.004	−0.01 (0.03)	.87	−0.01 (0.01)	.53

Abbreviations: DEXA, dual x-ray absorptiometry; HDL, high-density lipoprotein; LDL, low-density lipoprotein; SES, socioeconomic status.

^a^ The first half of the table used latent growth curve models, and the second half used path analysis with a single observed outcome variable. The second half was run using full information maximum likelihood and then again using listwise deletion, and the results were consistent.

**Figure.**
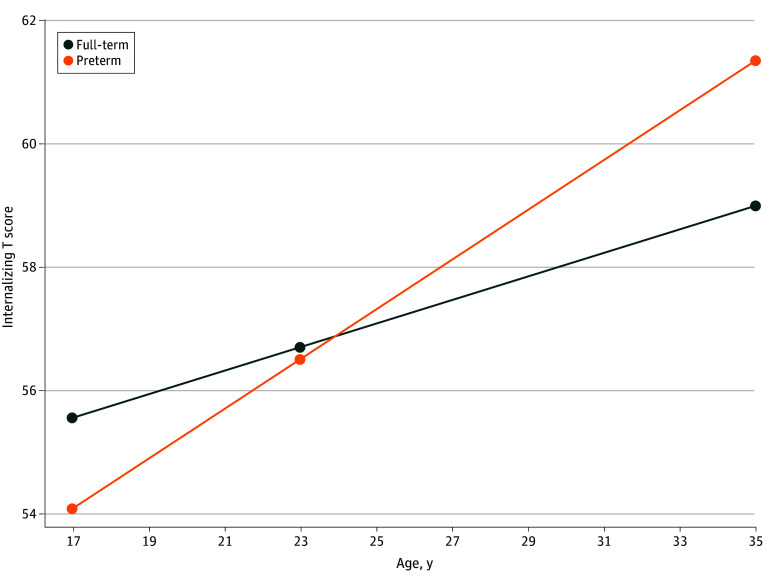
Latent Curve Model Results for Internalizing Trajectories in Adulthood by Medical Risk Levels Separate slopes are plotted based on the medical risk means of full-term vs preterm infants, but analyses used a continuous medical risk index.

### Physical Health

We used the same latent curve model for adulthood trajectories of systolic and diastolic blood pressure (DBP). Higher levels of medical risk were associated with higher systolic blood pressure (SBP) levels (β [SE], 7.15 [2.47]; *P* = .004), but not with DBP (Table 2). Next, we used path models to examine levels of cholesterol at age 35 years. A higher medical risk was associated with lower HDL (good) cholesterol (β [SE], −13.07 [4.4]; *P* = .003) and was not associated with LDL cholesterol. Across models, social protection and childhood SES covariates were not associated with levels or trajectories of cardiovascular risk.

Using path models to examine outcomes at age 35 years, higher medical risk severity was longitudinally associated with higher triglycerides (β [SE], 53.97 [24.6]; *P* = .03) but was not associated with hemoglobin A_1c_. Across models, social protection and childhood SES covariates were not associated with levels or trajectories of cardiovascular risk.

For inflammation and immune regulation, medical risk and social protection levels were not associated with C-reactive protein (CRP) or interleukin 6 (IL-6) levels. Higher childhood SES was associated with lower IL-6 levels (β [SE], −0.04 [0.02]; *P* = .01), but social protection was not associated with either outcome.

Using path models, we examined the android-to-gynoid fat ratio (higher ratio suggests fat is stored disproportionately in the abdominal region) and bone density T scores. Medical risk severity was associated with a higher android-to-gynoid fat ratio (β [SE], 0.22 [0.08]; *P* = .006) and lower bone density (β [SE], −1.14 [0.40]; *P* = .004). Social protection and SES childhood covariates were not associated with DEXA scan outcomes.

## Discussion

In this cohort study, US adults born preterm with higher early life medical risk had poorer psychological and physical health outcomes compared with full-term peers. Preterm individuals with more medical issues from birth to childhood had increased internalizing problems; SBP, HDL, and triglyceride levels; and central adiposity, along with lower bone density in the third decade of life.

Participants born preterm with early life medical issues showed higher adult internalizing problems, including anxiety, depression, withdrawal, and somatic complaints. Trajectory analyses indicated a faster increase in internalizing problems from ages 17 to 35 years compared with peers. A Norwegian study with very low birth weight (VLBW; under 1500 g) preterm individuals also found elevated internalizing problems from adolescence to young adulthood, along with increased externalizing symptoms.^26^ A meta-analysis from the Adults Born Preterm International Collaboration international research network revealed that the preterm group faced more internalizing but fewer externalizing issues.^27^ These findings, combined with our US cohort results, underscore the need to monitor psychological health as those born preterm age into adulthood.

Preterm-born adults with greater infant medical severity exhibited higher SBP, lower HDL levels, and elevated triglycerides. Similar findings have been reported in international cohorts.^28,29,30^ A systematic review and meta-analysis involving preterm and term-born children, adolescents, and adults from 8 countries found that preterm participants had significantly higher SBP than their term-born counterparts.^31^ Other studies found adults born preterm exhibit distinct ventricular structures that worsen with increased SBP.^32,33,34,35,36^ Together, this evidence highlights cardiometabolic risk in adults born preterm.

Adults born preterm in our study with higher infant medical severity showed lower bone mineral density than full-term peers. A Finnish study of 29-year-old adults born preterm with VLBW vs their same-sex full-term siblings found reduced bone density in the preterm group, not entirely explained by body size.^37^ Similarly, a Norwegian study of 25-to-28-year-old preterm VLBW adults and small for gestational age full-term adults indicated that both LBW groups exhibited lower bone density than controls, with the VLBW group having the lowest density. These results suggest an increased bone fracture risk for preterm individuals, consistent with findings from other global preterm cohorts.^38,39^

In the US, research on adult health outcomes of preterm individuals is limited. Most studies focus on the smallest 20% of premature infants, particularly those with very and extremely low birth weight (ELBW; below 1000 g).^40^ This narrow emphasis highlights the need for broader research on preterm births. A US study from the early 2000s on VLBW infants showed that their neurodevelopmental and growth issues persisted into young adulthood, leading to lower education, reduced physical capabilities, higher blood pressure, and poor respiratory function.^41,42,43^ International studies have confirmed these findings, showing higher rates of chronic health issues, lower educational success, and decreased adult independence in ELBW groups.^44,45,46,47,48,49,50^ Finnish registry data from adolescents to age 30 years revealed that preterm-born individuals faced increased risks of chronic multimorbidity.^18^ Similarly, a Norwegian study of 26-year-olds and 28-year-olds born VLBW reported worse general functioning and mental health compared with term-born peers.^51^

Many European cohorts have distinct characteristics compared with the US population, such as greater access to health services, more racial homogeneity, less socioeconomic disparity, and underrepresentation of high-risk groups.^48^ These disparities highlight the need for US-specific research on health outcomes for individuals born preterm within the US health care system and society.

Despite evidence of long-term psychological and physical issues, many health care professionals lack knowledge about and resources for this group. The absence of evidence-based clinical screening guidelines leads to missed opportunities for early intervention and preventive care. Research shows that those born preterm face increased risks for systemic disorders, including cardiovascular conditions,^14,34,52,53,54^ pulmonary dysfunction,^55^ kidney impairment,^56,57^ metabolic dysregulation,^28^ and mental health problems.^13,51^ This situation requires a life-course approach to effectively manage their health and implement targeted interventions.

Despite evidence of the long-term health implications of preterm birth, a substantial knowledge gap persists among US primary care clinicians. Many health care professionals remain unaware that they may be treating patients with a history of preterm birth due to the lack of routine inquiries about birth history in adult health care.^59^ This oversight supports the misconception that prematurity only matters in childhood. Futhermore, evidence-based clinical screening guidelines are needed for adults born preterm to prevent missed early intervention opportunities and tailored preventive care.

### Limitations

Several limitations must be considered when reflecting on this study’s findings. Recruiting participants from a high-performing NICU has significant implications. It ensured optimal neonatal care, likely influencing long-term health trajectories and survival rates, especially for high-risk infants. The facility’s involvement in clinical trials suggests participants had exposure to innovative treatments, impacting their health outcomes. These aspects, along with the lack of racial diversity within the cohort, should be carefully considered when interpreting study results and their generalizability to preterm populations.

Next, we consider the direct and indirect COVID-19 pandemic impacts. Historically, high participant retention rates have been achieved in our follow-up studies. In this study, only 73% of the original cohort participated at age 35 years, down from 84% at age 23 years. Factors like travel restrictions, masking, remote work, economic issues, and general concerns likely affected recruitment and measurement. The in-state COVID-19 lockdown lasted from March 2020 to July 2021, but various effects continued beyond that period.^58^

The third decade of life is also busy, as many balance personal and professional responsibilities, often raising children while focusing on careers. Taking time away from these duties is challenging. Some participants rejoined the study because their children were born preterm, valuing the findings, while others without such connections may have found participation less important. The small full-term sample size limited the power to assess effect modifications. Nonetheless, this 35-year prospective cohort offers multi-informant and multiple-method assessments, including medical records, molecular data, and various self-report measures, providing rich and unique data.

## Conclusions

In this cohort study, long-term outcomes following preterm birth revealed increased mental health, cardiometabolic, and body composition risks in adults born preterm compared with their full-term peers. These findings impact public health, health care delivery, and policy development. As the preterm-born population grows and ages, understanding their health needs is crucial for optimizing health outcomes and resources.

## Notes

Supplement 1.
eMethods.
**eTable 1.** Follow-up Studies for the RHODE Study by Age, Sample Size, and Funding Years **eTable 2.** Premature Cohort Demographics at Age 35 Years Follow-up **eFigure.** Bivariate Correlations Heatmap **eTable 3.** Model Fit Statistics

Supplement 2.
Data Sharing Statement
