# Supplementary material for: Psychological and Physical Health of a Preterm Birth Cohort at Age 35 Years
Source: JAMA Netw Open. 2025 Jul 22;8(7):e2522599. doi: 10.1001/jamanetworkopen.2025.22599 (PMC12284743; doi:10.1001/jamanetworkopen.2025.22599)
Supplement: Supplement 1. — eMethods. eTable 1. Follow-up Studies for the RHODE Study by Age, Sample Size, and Funding Years eTable 2. Premature Cohort Demographics at Age 35 Years Follow-up eFigure. Bivariate Correlations Heatmap eTable 3. Model Fit Statistics [file jamanetwopen-e2522599-s001.pdf]

Supplemental Online Content

D'Agata A, Eaton C, Smith T, et al. Psychological and physical health of a preterm birth cohort at age 35 years. *JAMA Netw Open.* 2025;8(7):e2522599. doi:10.1001/jamanetworkopen.2025.22599

eMethods.

eTable 1. Follow-up Studies for the RHODE Study by Age, Sample Size, and Funding Years

eTable 2. Premature Cohort Demographics at Age 35 Years Follow-up

eFigure. Bivariate Correlations Heatmap

eTable 3. Model Fit Statistics

This supplemental material has been provided by the authors to give readers additional information about their work.

© 2025 D'Agata A et al. *JAMA Network Open*.

eMethods

Medical risk:

The medical record was the source for birth and neonatal data. The Hobel was designed to assess perinatal factors in the mother and neonate. There are 51 prenatal items, 40 intrapartum items, and 46 neonatal items, each weighted according to assumed risk and then summed. The Neonatal Risk Score was used in this study. Construct and predictive validity were established in a series of studies[21]. Medical health status was classified as normal (no abnormalities), suspect (continued chronic respiratory problems, cardiac murmurs, referral for hearing, and orthopedic issues), or abnormal (asthma, allergies, diabetes, and/or autoimmune deficiencies). Neurological health status was classified as normal (no abnormalities), suspect (fine motor weakness, unilateral sensorineural hearing loss, uncorrected vision problems, atypical neurologic findings in tone, reflexes, gait, or movement with no specific diagnosis), or abnormal (cerebral palsy, blindness, deafness, shunted hydrocephalus, uncontrolled seizures, or attention-deficit/hyperactivity disorder[57,58]).

ASEBA:

ASEBA is a standardized assessment that measures strengths and behavioral, emotional, social, and thought problems in preschoolers through older adults. Adult Self-Report (ASR) is a 126-item questionnaire for adults aged 18-59. Youth Self-Report (YSR) is a 112-item questionnaire for youths aged 11-18. Responses for both instruments are rated on a 3-point scale: 0-Not True, 1-Somewhat/Sometimes True, and 2-Very True/Often True. T-scores were used in analyses for internalizing and externalizing problems for comparability across the youth and adult versions.

Social protection:

HOME subscales were emotional and verbal responsivity, encouragement of maturity, emotional climate, growth-fostering materials and experiences, provision for active stimulation, family participation in developmentally stimulating experiences, paternal involvement, and aspects of the physical environment. Items are scored as yes/no (yes 1⁄4 1, no 1⁄4 0) and summed for a total score.

Maternal involvement was assessed at age 4 years by having children and their mothers engaged in a spontaneous free-play session with age-appropriate toys. Examiners coded the videotape of the session using the Parent/Caregiver Involvement Scale, in which higher scores indicate greater parent involvement. At ages 8 and 12 years, maternal involvement was assessed in a session using Tangoes Puzzle™ pieces, where investigators asked the mother to "teach" her child to assemble a series of five puzzles. Examiners later coded the videotape of the session for behaviors of involvement, teaching, pacing, and appropriateness on a 5-point Likert-type scale, with higher scores indicative of greater maternal involvement.

Maternal control style was collected at ages 4- and 8-years using Playskool Pipeworks™ pieces in a problem-solving task. The mother and child were sequentially presented two completed models and enough Pipeworks pieces to replicate a table and wagon. The mother was asked to provide as much assistance as needed for her child to complete both tasks. Examiners later coded the videotape of the problem-solving session. At age 12 years, maternal control style was collected during the previously mentioned Tangoes Puzzle teaching segment. Coders rated supportive presence, quality of assistance, and maternal affect on 5-point Likert-type scales, with the higher summed score indicative of greater maternal control style. Examiners were blind to the prematurity status of participants and maintained an interrater reliability of 95% and above on measurement coding for both maternal involvement and maternal control assessments.

Recruitment and COVID-19. The tenth follow-up study took place during the COVID-19 pandemic, with recruitment for in-person visits starting in February 2020. However, these activities were paused from March 2020 until March 2021 due to the pandemic. Once they resumed in 2021, the study encountered further challenges from travel restrictions and increases in COVID-19 cases. Despite these obstacles, data collection continued until March 2024. The final sample included 143 participants: 126 completed in-person visits, while 18 had partial visits. Participants with partial visits completed only survey data and self-collected biospecimens; they did not attend in-person clinic visits. This adaptation underscores the study's ability to respond to the limitations imposed by the pandemic. Partial study visits were also completed for participants with severe sequelae from preterm birth.

Clinical Classifications:

Glycosylated hemoglobin was considered normal with values ≤5.7%, prediabetes with values of 5.8-6.4%, and diabetes with values of ≥6.5%. Lipid classifications were considered normal total cholesterol at ≤199 mg/dl and abnormal at ≥200 mg/dl; HDL for men was normal at ≥40 mg/dl and abnormal at ≤40 mg/dl; for women, it was normal at ≥50 mg/dl and abnormal at ≤50 mg/dl; LDL was optimal at ≤129 mg/dl and abnormal at high ≥130 mg/dl; triglycerides were normal at ≤149 mg/dl and abnormal at ≥150 mg/dl. DEXA adiposity for the android to gynoid ratio (A/G ratio) was interpreted such that abnormal levels were > 1.0 for men and > 0.8 for women. For DEXA, bone mineral density was interpreted such that T scores -1.0 or higher indicated normal levels.

© 2025 D'Agata A et al. *JAMA Network Open*.

Laboratory methods.

Hemoglobin A1c (HbA1c): Determination on Roche Cobas 6000 system based on turbidimetric immunoinhibition using hemolyzed whole blood or packed red cells (Roche Diagnostics, Indianapolis, IN). In sample, HbA1c reagent antibodies react specifically with glycated hemoglobin (HbA1c) and form soluble antigen-antibody complexes. Polyhaptens added to bind excess antibodies, resulting agglutinated complex measured turbidimetrically. Amount of HbA1c in sample inversely proportional amount of turbidity formed. Reported result is calculation of %HbA1c in total hemoglobin. Assay approved by FDA for clinical use and certified by National Glycohemoglobin Standardization Program for no interference by Hb C trait, Hb S trait, Hb E trait, elevated HbF, or Carbamyl-Hb. HbA_{1c} measurements of blood from long-term storage highly correlate to measurements from blood obtained before storage. Day-to-day variability at %A1c values of 4.4 and 10.6 are 1.9 and 1.5%.
Lipoprotein Profile: Determination of total cholesterol, triglycerides, high-density lipoprotein cholesterol (HDL-C) and low-density lipoprotein cholesterol (LDL-C) concentrations simultaneously performed on Roche Cobas 6000 system using reagents and calibrators from Roche Diagnostics (Indianapolis, IN). Assays approved by FDA for clinical use. BCH laboratory certified by CDC/BHLBI Lipid Standardization Program.
Total Cholesterol: Measured enzymatically, combining specificity of enzymatic reaction with peroxidase/phenol-4-aminophenazone indicator reaction. Cholesterol esters are hydrolyzed by cholesterol esterase to produce free cholesterol. In presence of oxygen and cholesterol oxidase, cholesterol oxidized to cholest-4-en-3-one and H_2O_2. Latter product reacts with dye to generate a quinoneimine dye. Intensity of generated color measured at 505 nm and directly proportional to concentration of cholesterol in measured sample. Lowest detection limit of this assay 3.86mg/dL; day-to-day imprecision values at concentrations of 76.2 and 276 mg/dL are 1,6 and 1.4%.
Triglycerides: Measured enzymatically with correction for endogenous glycerol. Preliminary reaction, endogenous glycerol phosphorylated in presence of glycerol kinase and ATP. Formed glycerol-3-phosphate oxidized to generate H_2O_2, reacting with 4-chlorophenol to produce oxidative product. Actual assay reaction triglycerides hydrolyzed by lipase mixture to generate glycerol and fatty acids. Similarly to preliminary reaction, glycerol phosphorylated by action of glycerol kinase and generated glycerol-3-phosphate oxidized to produce H_2O_2. Latter product reacts with dye to generate colored product. Intensity of generated color measured at 505 nm and directly proportional to concentration of triglycerides in measured sample. Lowest detection limit of assay 8.85 mg/dL and day-to-day imprecision values at concentrations of 104 and 261 mg/dL are 1.9 and 1.8%.
High Density Lipoprotein Cholesterol: Concentration determined using direct enzymatic colorimetric assay. Soluble complexes of non-HDL lipoproteins [low-density lipoproteins (LDL), very low-density lipoproteins (VLDL) and chylomicrons] and sulfated alpha-cyclodextrin-Mg++ form. Cholesterol component of HDL determined using polyethylene glycol (PEG)-modified cholesterol oxidase and esterase, which possess very limited reactivity with complexed apolipoprotein B-containing lipoproteins. Lowest detection limit of this assay 3.09 mg/dL and day-to-day imprecision values at concentrations of 9.48, 59.4 and 141 mg/dL are 2.2, 0.7 and 0.8%.
Low Density Lipoprotein Cholesterol: Determined by homogenous direct method from Roche Diagnostics (Indianapolis, IN). First reaction, specific detergent solubilizes all non-LDL lipoproteins. Enzymes cholesterol oxidase and cholesterol esterase react with non-LDL cholesterol without generation of color. Second reaction, another specific detergent solubilizes LDL so cholesterol component easily measured enzymatically, generating color reaction. Lowest detection limit of assay 3.87 mg/dL and day-to-day imprecision values at concentrations of 12.2, 315 and 530 mg/dL are 2.5, 1.9 and 2.0 %.
High Sensitivity C-Reactive Protein (hsCRP): Concentration determined with immunoturbidimetric assay on Roche Cobas 6000 system (Roche Diagnostics - Indianapolis, IN), using reagents and calibrators from Roche. Antigen-antibody reaction occurs between CRP in sample and anti-CRP antibody sensitized to latex particles and agglutination results. Antigen-antibody complex causes decrease transmitted light, detected spectrophotometrically, magnitude of change being proportional to concentration of CRP in sample. Assay approved by FDA for clinical use. High-sensitivity assay limit of detection of 0.15 mg/L. Day-to-day variabilities of assay at concentrations of 0.53 and 13.3 mg/L are 8.4 and 2.1%.
Glucose: Measured enzymatically on Roche Cobas 6000 system using Roche Diagnostics reagents (Indianapolis, IN). Hexokinase catalyzes phosphorylation of glucose by ATP, generating glucose-6-phosphate. Latter oxidized to 6-phosphogluconate in presence of NADP and glucose-6-phosphate dehydrogenase. Amount of NADPH formed during reaction directly proportional to amount of glucose present in sample and measured photometrically by increase in absorbance. Hexokinase method long been recognized as most specific method for determination of glucose. Assay approved for clinical use by FDA. Lowest detection limit of assay 2.0 mg/dL and day-to-day imprecision values at concentrations of 95.1 and 137 mg/dL are 1.1 and 1.2 %.
Fibrinogen: Concentration determined using immunoturbidimetric assay on Roche Cobas 6000 system (Roche Diagnostics - Indianapolis, IN), using reagents and calibrators from Kamiya Biomedical Co. (Seattle, WA). Antigen-antibody reaction occurs between fibrinogen in sample and anti-fibrinogen antibody, and agglutination results forming insoluble immune complex. Antigen-antibody complex causes increase in light scattering, detected spectrophotometrically, with magnitude of change being proportional to concentration of fibrinogen in sample. Day-to-day variabilities of assay at concentrations of 167.4, 323.6 and 554.1 mg/dL are 0.94, 1.06, and 1.50%.
Insulin: Measured by electrochemiluminescence immunoassay on Roche Cobas 6000 system (Roche Diagnostics, Indianapolis, IN). Biotinylated monoclonal insulin antibody and second monoclonal insulin antibody labeled with ruthenium are mixed with sample. Insulin in sample 'sandwiched' between antibodies, forming immunocomplex that is ruthenium-labeled and biotinylated. Streptavidin-coated magnetic microparticles added to reaction mixture to bind biotinylated antibody. Immunocomplexes magnetically

© 2025 D'Agata A et al. *JAMA Network Open*.

entrapped on electrode and unbound reagents, sample washed away. Chemiluminescent reaction electrically stimulated to generate light, intensity indirectly proportional to amount of insulin present in sample. Cross-reactivity of antibodies with proinsulin less than 0.05%, assay measures 'true' insulin. Assay FDA approved for clinical use. Lowest detection limit of assay 0.2 uU/mL and day-to-day imprecision values at concentrations of 6.85, 16.7 and 425 uU/mL are 4.9, 3.7 and 2.4%.

Dehydroepiandrosterone sulfate (DHEAS): Measured by competitive electrochemiluminescence immunoassay on Roche Cobas 6000 system (Roche Diagnostics, Indianapolis, IN). Biotinylated DHEAS antibody mixed with serum sample. DHEAS in sample binds to biotinylated antibody and forms immunocomplex. DHEAS derivative labeled with ruthenium and streptavidin-coated magnetic microparticles added to reaction mixture. Derivative fills rest of binding sites on biotinylated antibody and streptavidin particles interact with biotin on antibody. Immunocomplexes magnetically entrapped on electrode and unbound reagents and sample washed away. Chemiluminescent reaction electrically stimulated to generate light, intensity indirectly proportional to amount of DHEAS present in sample. Assay FDA approved for clinical use. Lowest detection limit of assay 0.1 ug/dL and run-to-run imprecision values at DHEAS concentrations of 93.2, 395 and 753 ug/dL are 2.5, 2.7 and 4%.

Interleukin-6 (IL-6): Measured by ultra-sensitive ELISA assay from R & D Systems, Minneapolis, MN. Assay employs quantitative sandwich enzyme immunoassay technique. Monoclonal antibody specific for IL-6 pre-coated onto microtitre plate. Addition of samples, standards, controls and conjugates to wells, IL-6 sandwiched between immobilized antibody and enzyme-linked antibody specific to IL-6. Upon addition of substrate, color generated proportional to amount of IL-6 present in sample. Assay has mean detectable dose of 0.031 pg/mL, and day-to-day variabilities of assay at concentrations of 0.53, 2.75 and 5.58 pg/mL are 10.8, 4.92 and 3.9%.

Brain-derived Neurotrophic Factor (BDNF): Measured by ELISA assay from R&D Systems (Minneapolis, MN). Assay employs quantitative sandwich enzyme immunoassay technique. Monoclonal antibody specific for BDNF pre-coated on microtitre plate. Addition of samples, standards, and controls to wells and plate is incubated. Conjugate added, second BDNF monoclonal antibody linked to enzyme. BDNF in samples sandwiched between immobilized antibody on microtitre plate and enzyme-linked antibody. Plate washed to remove unbound compounds. Upon substrate addition, color generated proportional to amount of BDNF present in sample. Assay possesses sensitivity of .997 pg/mL and run-to-run imprecision at BDNF concentrations of 98.8, 296 and 594 pg/mL are 7.2, 4.3 and 4.7%.

© 2025 D'Agata A et al. *JAMA Network Open*.

eTable 1. Follow up studies for the RHODE Study by age, sample size, and funding years.									
Birth[59-61]	3 m[62,63]	9 m[64]	18 m[65]	4 y[66-68]	8 y[69,70]	12 y[71]	17 y[72,73]	23 y[74,75]	35 yrs.
215	215	215	215	184	188	186	180	180	143
1985-1989	1985-1989	1985-1989	1985-1989	1989-1993	1994-1997	1998-2002	2003-2008	2008-2014	2019-2023

© 2025 D'Agata A et al. *JAMA Network Open*.

eTable 2. Premature cohort demographics at age 35 follow-up.

	Preterm (N = 115)	Full-term (n = 29)
Age – M(SD)	34.23 (1.26)	35.29 (1.41)
Sex		
Male	41.7%	41.4%
Female	58.3%	58.6%
Race / Ethnicity		
American Indian	.9%	0%
Asian	.9%	0%
Black	9.6%	10.3%
White	84.3%	82.8%
Multiple	6.1%	10.3%
Hispanic or Latino	4.3%	6.9%
Marital Status		
Single	40.0%	37.9%
Cohabitating	16.5%	10.3%
Married	34.8%	44.8%
Separated/Divorced	8.7%	6.9%
Education		
HS or GED	18.3%	24.1%
Partial College	23.5%	37.9%
College	36.5%	27.6%
Graduate +	21.7%	10.3%
Employed	82.3%	65.5%
SES		
High	19.1%	13.8%
Moderate high	41.7%	34.5%
Average	23.5%	20.7%
Moderate low	12.2%	17.2%
Low	3.5%	13.8%

© 2025 D'Agata A et al. *JAMA Network Open*.

eFigure. Bivariate correlations heatmap.

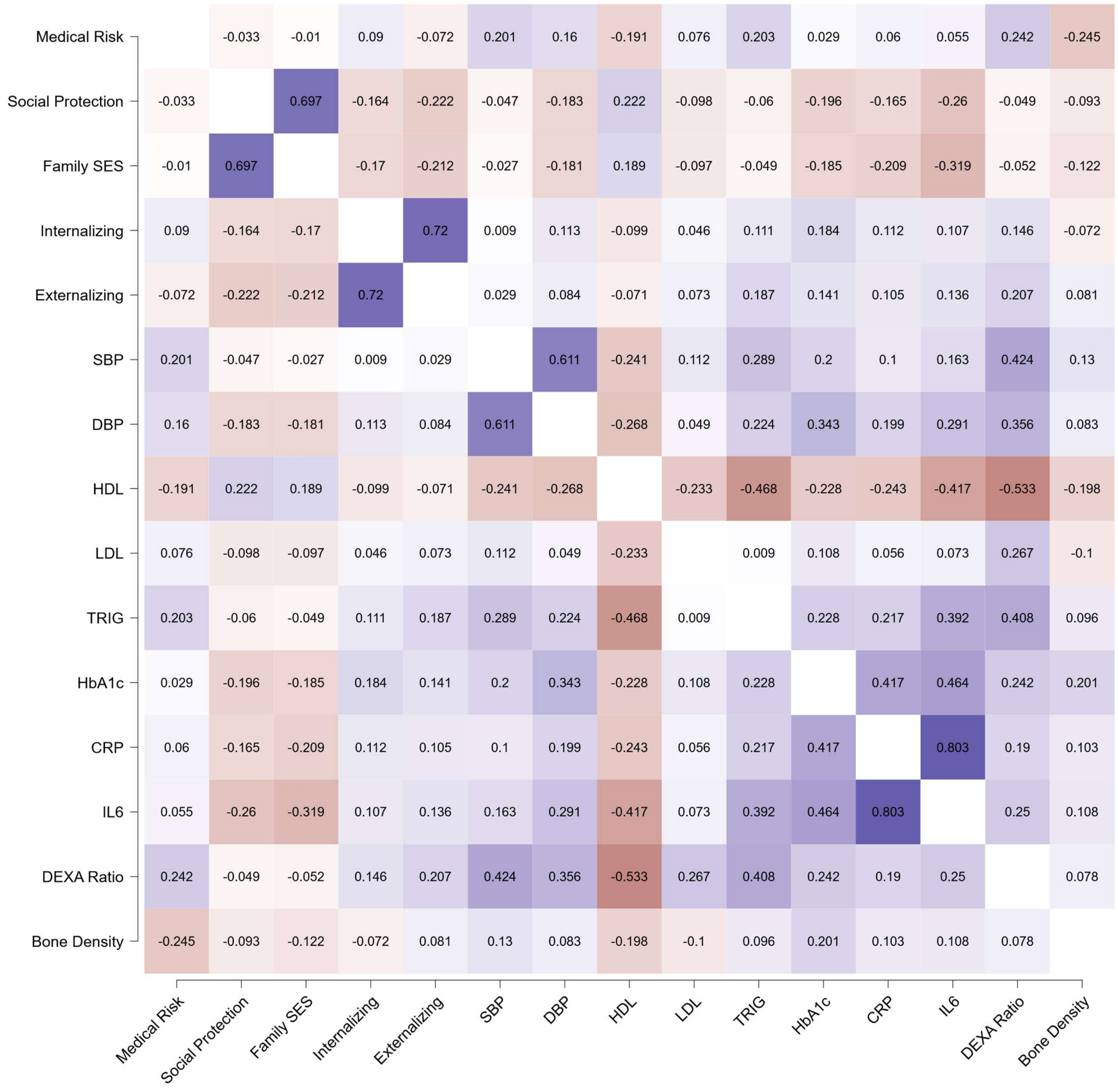

© 2025 D'Agata A et al. *JAMA Network Open*.

eTable 3. Model fit statistics.

Model Outcome	χ^2 (df)	p	RMSEA	95% CI	SRMR
Internalizing	2.65 (4)	.617	.000	.000, .086	.018
Externalizing	9.51 (4)	.049	.080	.003, .148	.039
Systolic Blood Pressure	3.93 (5)	.559	.000	.000, .084	.048
Diastolic Blood Pressure	9.98 (6)	.125	.056	.000, .115	.072

eTable 3 References.

59. Boukydis CFZ, Lester BM. Infant crying, risk status and social support in families of preterm and term infants. *Early Development and Parenting*. 1998/03/01 1998;7(1):31-39. doi:https://doi.org/10.1002/(SICI)1099-0917(199803)7:1<31::AID-EDP161>3.0.CO;2-9

60. McGrath M, Boukydis CFZ, Lester BM. Determinants of maternal self-esteem in the neonatal period. *Infant Mental Health Journal*. 1993/03/01 1993;14(1):35-48. doi:https://doi.org/10.1002/1097-0355(199321)14:1<35::AID-IMHJ2280140104>3.0.CO;2-R

61. Lester BM, Boukydis CFZ, LaGasse L. Cardiorespiratory reactivity during the Brazelton Scale in term and preterm infants. *Journal of Pediatric Psychology*. 1996;21(6):771-783. doi:10.1093/jpepsy/21.6.771

62. Bigsby R, Coster W, Lester BM, Peucker MR. Motor behavioral cues of term and preterm infants at 3 months. *Infant Behavior and Development*. 1996/07/01/ 1996;19(3):295-307. doi:https://doi.org/10.1016/S0163-6383(96)90030-2

63. Lester BM, Zachariah Boukydis CF, Garcia-Coll CT, Hole W, Peucker M. Infantile colic: Acoustic cry characteristics, maternal perception of cry, and temperament. *Infant Behavior and Development*. 1992/01/01/ 1992;15(1):15-26. doi:https://doi.org/10.1016/0163-6383(92)90003-O

64. Meyer EC, Zeanah CH, Boukydis CFZ, Lester BM. A clinical interview for parents of high-risk infants: Concept and applications. *Infant Mental Health Journal*. 1993/09/01 1993;14(3):192-207. doi:https://doi.org/10.1002/1097-0355(199323)14:3<192::AID-IMHJ2280140305>3.0.CO;2-R

65. Lester BM, Boukydis CF, Garcia-Coll CT, et al. Developmental outcome as a function of the goodness of fit between the infant's cry characteristics and the mother's perception of her infant's cry. *Pediatrics*. Apr 1995;95(4):516-21.

66. McGrath MM, Sullivan, M.C. Maternal interaction patterns and preschool outcomes in high-risk children. *Nursing Research*. 1998;47(6):309-317.

67. McGrath M, Sullivan, M. C., Brem, F., Rocherolle, K. Mastery motivation and cognitive development in 4-year-old children born at various degrees of medical risk. *Journal of Pediatric Nursing*. 1995;10(5):287-295. doi:https://doi.org/10.1016/S0882-5963(05)80046-6

68. McGrath MM, Sullivan M. Testing proximal and distal protective processes in preterm high-risk children. *Issues in Comprehensive Pediatric Nursing*. 2003/01/01 2003;26(2):59-76. doi:10.1080/01460860390197835

69. Sullivan MC, Margaret MM. Perinatal morbidity, mild motor delay, and later school outcomes. *Dev Med Child Neurol*. Feb 2003;45(2):104-12.

70. McGrath M, Sullivan M. Birth weight, neonatal morbidities, and school age outcomes in full-term and preterm infants. *Issues Compr Pediatr Nurs*. Oct-Dec 2002;25(4):231-54. doi:10.1080/01460860290042611

71. Sullivan MC, McGrath MM, Hawes K, Lester BM. Growth trajectories of preterm infants: Birth to 12 years. *Journal of Pediatric Health Care*. 2008;22(2):83-93. doi:10.1016/j.pedhc.2007.02.008

72. Sullivan MC, Miller RJ, Msall ME. 17-year outcome of preterm infants with diverse neonatal morbidities: part 2, impact on activities and participation. *Journal for specialists in pediatric nursing : JSPN*. Oct 2012;17(4):275-87. doi:10.1111/j.1744-6155.2012.00339.x

73. Sullivan MC, Msall ME, Miller RJ. 17-year outcome of preterm infants with diverse neonatal morbidities: Part 1, impact on physical, neurological, and psychological health status. *Journal for Specialists in Pediatric Nursing*. 05/29 2012;17(3):226-241. doi:10.1111/j.1744-6155.2012.00337.x

74. Sullivan MC, Winchester SB, Bryce CI, Granger DA. Prematurity and perinatal adversity effects hypothalamic-pituitary-adrenal axis reactivity to social evaluative threat in adulthood. *Developmental psychobiology*. Dec 2017;59(8):976-983. doi:10.1002/dev.21570

75. Sullivan MC, Winchester SB, Msall ME. Prematurity and cardiovascular risk at early adulthood. *Child Care Health Dev*. Jan 2019;45(1):71-78. doi:10.1111/cch.12616

© 2025 D'Agata A et al. *JAMA Network Open*.
