# Supplementary material for: Psychological and Physical Health of a Preterm Birth Cohort at Age 35 Years
Source: JAMA Netw Open. 2025 Jul 22;8(7):e2522599. doi: 10.1001/jamanetworkopen.2025.22599 (PMC12284743; doi:10.1001/jamanetworkopen.2025.22599)
Supplement: Supplement 2. — Data Sharing Statement [file jamanetwopen-e2522599-s002.pdf]

Data Sharing Statement

D'Agata. Psychological and Physical Health of a US Preterm Birth Cohort at 35 Years. *JAMA Netw Open*. Published July 22, 2025. doi:10.1001/jamanetworkopen.2025.22599

Data
Data available: No

Additional Information
Explanation for why data not available: Sample could be identified.
